# CancelRx implementation: Observed changes to medication discontinuation workflows over time

**DOI:** 10.1016/j.rcsop.2022.100108

**Published:** 2022-01-25

**Authors:** Taylor L. Watterson, Sara E. Hernandez, Jamie A. Stone, Aaron M. Gilson, Edmond Ramly, Michelle A. Chui

**Affiliations:** a University of Wisconsin-Madison School of Pharmacy, Madison, WI, USA; b University of Wisconsin School of Medicine and Public Health, Madison, WI, USA; c University of Wisconsin-Madison College of Engineering, Madison, WI, USA

**Keywords:** Health IT, Workflow, Medication discontinuation, CancelRx

## Abstract

**Introduction:**

When patients are seen in an ambulatory outpatient clinic, such as their primary care provider's office, the prescriber often stops or discontinues medications. Although medication discontinuations are documented in the clinic's health record, this information may not be communicated to the pharmacy. Within the last decade, CancelRx has attempted to address this issue by sending a message from the clinic to the pharmacy when a medication has been discontinued or changed.

**Objectives:**

This project studied pharmacy medication discontinuation workflows and pharmacists' perspectives at 3 UW Health outpatient pharmacies before and after implementation of CancelRx.

**Methods:**

CancelRx was implemented at UW Health in October 2017. Pharmacists from 3 outpatient pharmacies were observed at 3 distinct time points. The research team conducted 9 observations 3-months before CancelRx implementation (July 2017). Additionally, 9 observations were completed at 3-months after CancelRx implementation (January 2018) and at 9-months after CancelRx implementation (July 2018). Collective case study and comparative workflow modeling were used in this study. Observation field notes were deductively coded and aggregated to determine task frequency, occurrence, and patterns using an interpretivist theoretical approach.

**Results:**

During the study, 106 medication discontinuation instances (referred to as cases) were observed; 28 cases 3-months prior to CancelRx, 59 cases 3-months after CancelRx, and 16 cases 9-months after CancelRx. Medication discontinuation tasks aligned with the predetermined workflow: receiving and investigating the discontinuation messages, matching the message to the medication in the patient's profile and discontinuing it, documenting and communicating the message to others as necessary. After implementing CancelRx, the workflow changed as most pharmacists eliminated the investigating and documenting tasks.

**Conclusions:**

This study provided insight into the medication discontinuation workflow in community pharmacies, especially after implementing CancelRx. Organizations are recommended to proactively consider the implications for novel health information technology before implementation to anticipate workflow and pharmacy practice changes and improve acceptance and effectiveness.

## 1 Introduction

When patients are seen in an outpatient, ambulatory clinic, such as their primary care physician for an annual physical, their prescriber may stop or discontinue medications. Medications may be discontinued for a variety of reasons, including therapy changes, dose changes, completion of therapy, or adverse drug events and allergies. Although providers document medication discontinuations in their clinic's Electronic Health Record (EHR), when this information is not communicated to the pharmacy, there is a high likelihood of inaccurate medication lists and increased potential to fill inappropriate medications. In 2012, a study found that up to 5% of medications discontinued by prescribers were later dispensed to patients, with 34% meeting the high-risk criteria for potential adverse drug events.^1^

Historically, notifications about discontinued medications could be communicated to the pharmacy in a variety of ways, including telephone calls from clinic staff, faxed reports, notes attached to new prescriptions, and patient self-reports. After the pharmacy received the notification, a staff member or pharmacist needed to manually document the information in the pharmacy's dispensing platform to block future fills. These unstandardized processes required time and effort from both clinic and pharmacy staff members who had to prioritize amongst other clinical tasks.

## 2 CancelRx

Within the last decade, a health information technology (health IT) called CancelRx has emerged that attempts to address the problem of medication discontinuation messages.^2^ Like its name suggests, CancelRx is an IT function that automatically transmits an electronic message from the clinic to the pharmacy when a medication has been stopped, discontinued, or changed. The health IT follows the same channels as a new electronic prescription and, in some instances, can automatically match the cancellation message to the correct patient and medication at the community pharmacy, which reduces the work for the pharmacy staff and potentially improves efficiency and accuracy of communicated information.

In 2017, a large e-prescribing vendor, SureScripts, removed the financial restraints on the use of CancelRx. Previously a barrier to widespread implementation, CancelRx use spread with SureScripts acting as a third-party mediator that communicated messages between the clinic EHR and pharmacy dispensing platforms. Although there has been research to demonstrate how CancelRx affects health system outcomes, such as reducing medication list discrepancies, little is known about how this novel health IT affected the medication discontinuation workflow for pharmacy staff and how these processes changed over time after implementation.^3^^,^^4^

This project compared pharmacy medication discontinuation workflows before and after CancelRx implementation at three UW Health outpatient pharmacies. Secondary goals were to investigate the role of health IT (i.e., CancelRx) in pharmacy workflows and ascertain pharmacists' overall perspectives.

## 3 Methods

### 3.1 Study design

To characterize the medication discontinuation workflows before and after the implementation of CancelRx, this study used interpretivism as a theoretical framework, a collective case study methodology, and a comparative workflow modeling to compare tasks.^5^, ^6^, ^7^, ^8^, ^9^, ^10^ Interpretivism provided the opportunity to assess the specific steps in the medication discontinuation workflow and the meaning pharmacists attributed to the messages they received.^5^^,^^6^ The collective case study methodology allowed for in-depth assessment and description of the medication discontinuation workflow within a clearly identifiable and bounded system, the pharmacist, and required analyzing the observations to generate patterns and meaning.^7^^,^^8^ Comparative workflow modeling allowed for systematically comparing task occurrence using a single process of medication discontinuation as the basis for comparison at three points in time.^9^^,^^10^

### 3.2 Setting

#### 3.2.1 Sampling

Prior to data collection, this study was approved by the University of Wisconsin-Madison Institutional Review Board. The study team met with the head of outpatient pharmacy services at UW Health to facilitate stakeholder buy-in and identify a convenience sample of pharmacists across 3 UW Health outpatient pharmacies. The study team worked with the head of outpatient pharmacy services to schedule meetings with the pharmacy managers and obtained permission to recruit pharmacists and collect data.

### 3.3 Data collection

**Fig. 1:**
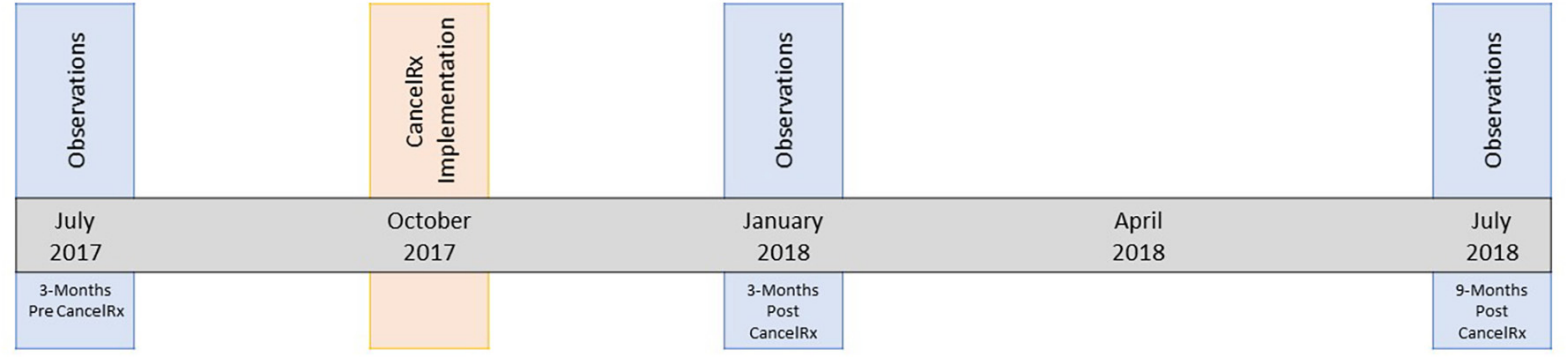
Data collection timeline. Data was collected during a one-year timeline surrounding CancelRx implementation (October 2017). Obercations were conducted at 3-Months Pre CancelRx (July 2017), 3-Months Post CancelRx (January 2018) and 9-Months Post CancelRx (July 2018). Each observation period consisted of 9 obervations (3 pharmacists at each of 3 pharmacies).

CancelRx was implemented at UW Health as part of a system-wide EHR upgrade in October 2017. The medication discontinuation workflow was observed at 3 unique time points: 3-months before CancelRx implementation (July 2017), 3-months after CancelRx implementation (January 2018), and 9-months after CancelRx implementation (July 2018) (represented in Fig. 1). During each data collection time period, a research team member (TLW) conducted 3-h long observations with 3 pharmacists at each of 3 pharmacies. In general, the observations captured pharmacist communications with pharmacy staff, patients, and healthcare providers, pharmacist interactions with technology and health IT, and the medication discontinuation workflow.^7^ The focus of pre-intervention data collection was to gather baseline information about processes, workflow, and workload surrounding medication discontinuations. The focus of the 3-month post-CancelRx data collection was to assess the adoption and impact of CancelRx in the pharmacy work systems shortly after its implementation. The 9-month post-CancelRx data collection focused on determining the sustainment and maintenance of changes to the medication discontinuation workflow and work systems.^11^ Observations occurred at various times of the day and various days of the week to account for variability in staffing or pharmacy business.

After conducting the observations, the researcher (TLW) transcribed their field notes and included a brief reflection about the observation. Field notes were stored on a secure server and verified to ensure no identifiable information was captured.

### 3.4 Data analysis

At the first stage of comparative workflow modeling, after data collection concluded, the researchers (TLW, JAS, MAC) gathered an interprofessional team of clinicians (PK, MS), scientists (TS, RB), and systems engineers (ER) with expertise in implementation science and human factors. Together, the team constructed a medication discontinuation process map detailing the series of events that occurred from the time a medication was discontinued in the clinic to the hand-off of information between clinic and pharmacy (either manually or via CancelRx) and the processing of the information at the community pharmacy (as well as the many opportunities for vulnerabilities or “failures”).^12^^,^^13^

At the next stage of the comparative workflow modeling, researchers (TLW, SH) focused on the components of the process map occurring at the community pharmacy and generated a workflow model encompassing all the pharmacists' tasks required to discontinue a medication.

Next, in the comparative workflow modeling, workflow tasks were used as the schema to conduct a deductive content analysis of the observation field notes (each task compromised one code).^8^^,^^14^ Each instance of a medication discontinuation identified in the field notes was considered a “case” comprised of workflow tasks. Coding for each discrete workflow task allowed the research team to systematically identify and compare the occurrence and variation of the tasks over time. Two researchers (TLW, SH) independently coded the field notes using Microsoft Word's “comment” function. The researchers compared their coding to identify discrepancies (none noted) and discuss emerging patterns.

The content analysis findings were shared with the interprofessional team to discuss patterns or relationships within the workflow. Analysis utilized the interpretivism theoretical philosophy, specifically symbolic interactionism, to identify the value pharmacists attributed to medication discontinuation messages and compare workflow over time.^5^

## 4 Results/findings

### 4.1 First stage: general medication discontinuation process map and workflow tasks

**Fig. 2:**
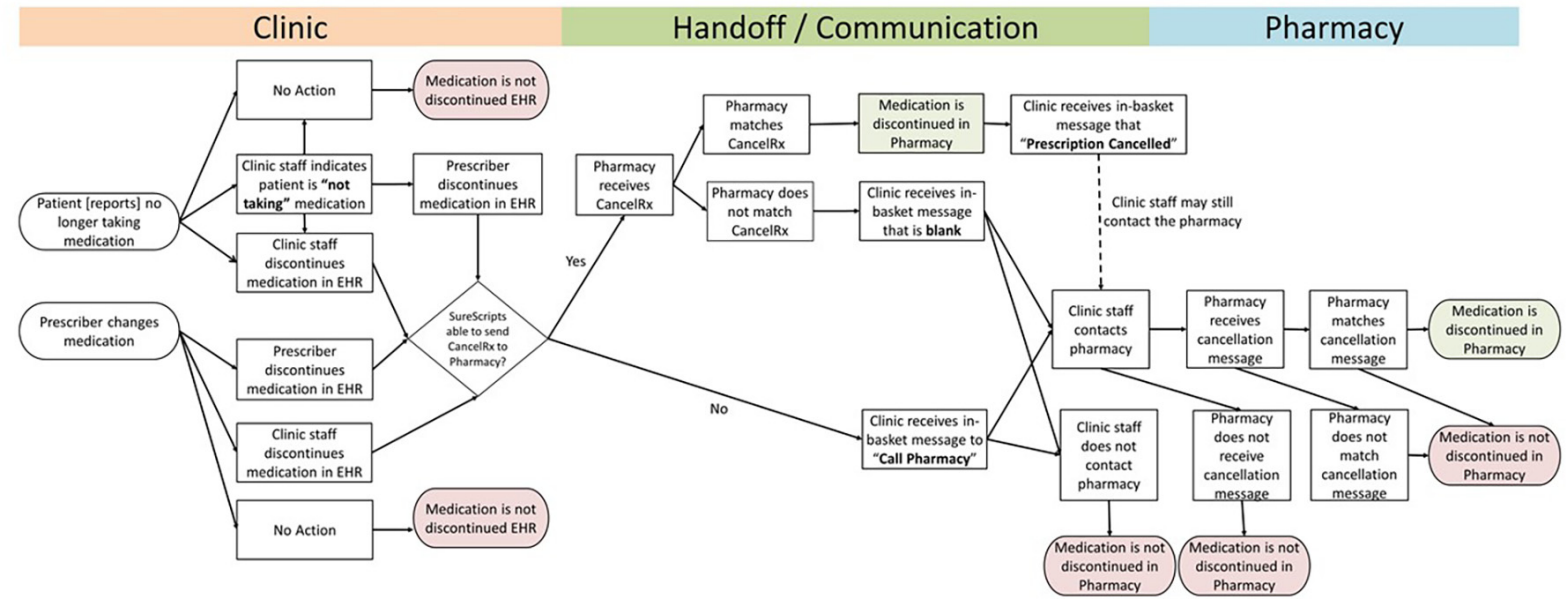
Medication discontinuation process map. Process map created by interprofessional research team. Displays medication discontinuation process from prescriber at clinic's office to successful discontinuation (green) at the community pharmacy and all potential failures (red).

The interprofessional team of clinicians, pharmacists, and researchers created a medication discontinuation process map (Fig. 2).

The team further detailed a pharmacy workflow with six discrete tasks:

(1) **Receives:** An actor (either the pharmacist or another member of the pharmacy staff) receives information that a patient's medication is discontinued. This could be via a phone call or fax from clinic staff, a patient report identified while conducting a review of the patient's profile in either the pharmacy dispensing system or the EHR, a note on a patient's prescription, or a CancelRx.
(2) **Investigates:** An actor (either the pharmacist or pharmacy staff member) further investigates the medication discontinuation message. This could involve looking into a patient's medication profile or health record, calling the clinic, asking the patient questions, or even asking another staff member for help.
(3) **Matches:** The actor (either the pharmacist, pharmacy staff member, or CancelRx) matches the medication from the discontinuation message to the appropriate medication and/or prescription in the patient's profile in the dispensing platform.
(4) **Discontinues:** The actor (either the pharmacist, pharmacy staff member, or CancelRx) discontinues the appropriate medication in the patient's profile in the pharmacy dispensing platform. This prevents the prescription from being filled or refilled in the future.
(5) **Documents:** The actor (either the pharmacist or pharmacy staff member) documents details regarding the medication discontinuation in the pharmacy dispensing platform (e.g., the date the medication was discontinued, who discontinued the medication, or a reason for discontinuation if known, etc.).
(6) **Communication:** The actor (either the pharmacist, pharmacy staff member, or CancelRx) communicates that the medication has or has not been discontinued. This communication may be to the patient, other pharmacy staff members, or back to the originating clinic that sent the discontinuation message.

### 4.2 Second stage: medication discontinuation comparative workflow modeling

The research team observed 28 distinct medication discontinuation cases at 3-months pre-CancelRx, 59 discontinuation cases 3-months post-CancelRx, and 16 discontinuation cases 9-months post-CancelRx. Table 1 details the occurrence and frequency of workflow tasks at the three time periods. A single task could constitute an entire case in some instances (e.g., a pharmacist communicates a medication discontinuation with a patient at the point of pick-up).

**Table 1:** Medication Discontinuation Comparative Workflow Model.

Task	Time Period					
	3-Months Pre CancelRx		3-Months Post CancelRx		9-Months Post CancelRx	
	*n* = 28 cases		*n* = 59 cases (48 via CancelRx)		*n* = 16 cases (10 via CancelRx)	
	Number Cases Task Occurred	% Cases Task Occurred	Number Cases Task Occurred	% Cases Task Occurred	Number Cases Task Occurred	% Cases Task Occurred
Receives	13	46%	55	93%	12	75%
Investigates	25	89%	27	46%	9	56%
Matches	13	46%	48	81%	10	63%
Discontinues	11	39%	48	81%	10	63%
Documents	5	18%	4	8%	1	6%
Communicates	7	25%	2	3%	1	6%

#### 4.2.1 3-months before CancelRx implementation

Before CancelRx implementation, pharmacists were the primary actors responsible for the medication discontinuation workflow. Although the health system's policy allowed technicians and other staff members to receive and execute discontinuation messages, the observed pharmacists indicated that the messages were usually directed to them.

Medication discontinuation often follows a linear process established in the general workflow model. Discontinuation messages frequently were received either via patient statements, such as “I'm not taking this” or “My doctor changed this medication,” prompting the pharmacist to investigate (5 of 13 total cases observed). Occasionally, pharmacists would receive discontinuation messages from clinic staff via phone calls or notes added to new prescriptions (3 of 13 cases). Pharmacists would often identify discontinuations while dispensing prescriptions during routine profile reviews (5 of 13 cases). The pharmacist investigated medication discontinuation by reviewing the patient's pharmacy profile or navigating to the health system's EHR.

The investigation task often occurred in tandem with the matching task. While the pharmacist researched the patient profile, they manually matched the discontinuation message to the appropriate prescription. While in the patient's medication profile, they were also able to discontinue the desired prescription by clicking a “Deactivate” button. After discontinuing a medication, they were presented with an optional text box to document any notes or comments. However, there were only 5 cases that included documentation in the pre-period (18% of cases).

Finally, the pharmacist would most commonly communicate the medication discontinuations with either the patient or other pharmacy staff members (6 cases and 1 case, respectively). Pharmacists would leave notes for other staff members to utilize at the point-of-sale (e.g., therapy change, previous dose discontinued) to discuss with the patient at the time of consultation. To illustrate this entire process, an exemplar medication discontinuation case is presented in Table 2.

**Table 2:** Exemplar medication discontinuation case 3-months pre-CancelRx (pharmacist 6).

Task	Time period 3-months pre CancelRx
Receives	The pharmacist looks at a patient's medication profile on the computer screen. The pharmacist states the patient was just prescribed Cresemba [Isavuconazole;] however, they had previously filled voriconazole.
Investigates	The pharmacist explains to the researcher these medications are in the same class and are not usually taken together. The pharmacist shows observer how they can pull up the health system's EHR to verify which medication the patient should be taking. The pharmacist opens EHR and locates the patient's profile. The pharmacist clicks on the search field in the upper right-hand corner of the patient's profile (indicated by a magnifying glass icon). The pharmacist types “Voriconazole” into the field, and a sidebar appears highlighting all the instances in the patient's profile where “voriconazole” appears. The pharmacist clicks on the first item in the list, a provider note. The pharmacist reads the note and states that the voriconazole is being discontinued and replaced by Cresemba.
Matches	The pharmacist states, “Then what I go do is deactivate… [the discontinued medication].” The pharmacist toggles back to the patient's profile in the pharmacy dispensing platform and clicks to the row indicating the voriconazole prescription.
Discontinues	Once selected and highlighted, the pharmacist clicks on the “Deactivate” button at the bottom of the page.
Documents	A pop-up window appears in the center of the screen with an open textbox. The pharmacist types, “On Cresemba per HL” [HL is the abbreviation of the EHR platform used]. Before hitting submit, the pharmacist adds a few words to the beginning of the note, which the observer does not see.
Communicates	N/A

#### 4.2.2 3-months post CancelRx implementation

When CancelRx was implemented, the health system administration formally decided only pharmacists would handle and address CancelRx messages instead of technicians and other staff who could discontinue medications in the pre CancelRx period. During the 3-month post-CancelRx observations, 59 medication discontinuations were observed, with 48 of these messages received via CancelRx (81% of cases). Because the CancelRx messages traveled through SureScripts, the same third-party vendor used to communicate new electronic prescriptions (e-prescriptions), CancelRx was able to automatically “match” the discontinued prescription to the previous record using an e-prescription order identifier. If CancelRx matched the prescription, it instantly deactivated the prescription record in the dispensing platform and sent a notification to the originating prescriber. The Receive, Match, Discontinue, and even Communication tasks were completed instantaneously by CancelRx.

In 4 cases, CancelRx did not automatically match the prescription, and the pharmacists manually matched and discontinued the medications in the patients' profiles. Pharmacists explained this occurred if the prescription had been transferred to a different pharmacy and was no longer in their system or if the cancellation message was for a prescription written more than five years prior. The pharmacist could also indicate “No Match Found” if there were no prescription records available for the cancellation message, and CancelRx would notify the prescriber that the pharmacy was unable to discontinue the medication and to follow up as needed.

When pharmacists opened the CancelRx message, they sometimes investigated the case by reviewing the patient's medication profile or EHR (22 of 48 cases; 46% of messages were received via CancelRx). For example, one pharmacist explained she checked the patient's EHR every time she received a CancelRx and asked herself, “Is it really canceled? Or are they [the prescriber] just removing it from the medication list and going to be sending a new prescription and renewing? I have to go into [the EHR] to find out.”

However, in over half of the CancelRx cases, the pharmacists did not “investigate” medication discontinuation messages. Upon probing, pharmacists felt they often did not need to check the patient's profile because the CancelRx messages were considered “low-value” – that is, for acute medications (e.g., antibiotic), had no refills (such as a Schedule II controlled medication), or were written over a year ago and no longer valid. Pharmacists also stated they were sometimes familiar with the patient or had a recollection of the patient's history or medication changes and did not need to investigate the patient's profile.

After any further investigation or matching, the pharmacist was required to attest to viewing the CancelRx message. Once confirmed, the message would disappear. If desired, the pharmacist could navigate to the patient's profile to add documentation notes or communications for other staff members (occurred in 3 cases).

During the observations, many pharmacists cited frustration with the sheer number of CancelRx messages, and they often contained “low-value” information. One pharmacist stated the messages were “meaningless,” and they never received training on how to handle them. Another pharmacist noted that the CancelRx messages did not contain a “reason why the medications are canceled, but that would be nice.” An exemplar case from the 3-month post-CancelRx time period is presented in Table 3.

**Table 3:** Exemplar medication discontinuation case 3-months pre-CancelRx (pharmacist 2).

Task	Time period: 3-months post CancelRx
Receives	The pharmacist navigates to the “Inbound Communication Queue,” where new messages and e-prescriptions from prescribers are received. There are over one dozen items in the queue (indicated by rows in a table). The pharmacist double clicks on a line/message, and the CancelRx screen appears.
Investigates	The pharmacist states that if there is something they “need to look at,” they can click on the patient's name and view the patient profile. The pharmacist states the prescription was for an urgent antibiotic, and it “makes sense” to be discontinued.
Matches	The CancelRx automatically matched the message to a prescription in the system. This is indicated because two rows of text are completed on the CancelRx screen. One row displays the received CancelRx message, and the second row displays the associated prescription found in the pharmacy record. [not observed in this exemplar, but if CancelRx could not match the prescription, the second row would be blank until the pharmacist manually matched the prescription].
Discontinues	The pharmacist clicks a button on the bottom left, “Remove from ICQ” [Inbound Communication Queue]. A pop-up appears asking, “Are you sure you want to remove from the inbound communication queue?” with the prompts “Yes” and “No.” They click “Yes,” and the pop-up screen and CancelRx message disappear. The pharmacist is returned to the previous queue and clicks on another order [process repeats].
Documents	N/A
Communicates	N/A [not observed, but clinicians on the research team (PK, MS) confirmed that CancelRx automatically sends a notification back to the clinic regarding the successful discontinuation]

#### 4.2.3 9-months post CancelRx implementation

Nine months after CancelRx implementation, the workflow did not change significantly. The team observed 16 medication discontinuation cases. Compared to earlier time periods, the fewer medication discontinuations may have been due to a general decline of messages sent overall or the pharmacists' increased time dedicated to administering flu vaccines during the influenza season. The medication discontinuation workflow remained the responsibility of pharmacists. However, technicians were becoming more involved in responding to errors or intervening when technical problems occurred (e.g., difficulties with billing prescriptions once they were discontinued or when prescribers accidentally discontinued prescriptions). Pharmacists stated the CancelRx messages were low on their priority list because they knew that the system automatically discontinued messages when it identified matches. Consequently, they had assurance a prescription would likely not be inadvertently dispensed before addressing the messages.

After 9 months, CancelRx was the primary way medication discontinuation messages were communicated to the pharmacy (10 cases, 63%). At that time, pharmacists rarely investigated CancelRx messages or referred to patient profiles (3 cases out of 10, 30% of all CancelRx cases). Pharmacists would occasionally explain their decision, indicating that a prescription was over a year old or filled for an acute condition.

As before, discontinuation message matching and discontinuing was primarily accomplished via CancelRx. At one pharmacy, a pharmacist and technician cited frustrations with CancelRx because once the IT automatically matched and discontinued a prescription, it was extremely difficult to reverse or “undo.” They shared an example of a frequent situation where a patient forgot her prescription insurance card but opted to pay cash for the medication. The pharmacy agreed that they would attempt to rebill her insurance once she returned with her card, adjudicate the claim, and refund the money she paid out-of-pocket. However, before the patient could return with her insurance card, the pharmacy received a CancelRx message that discontinued the prescription. The discontinuation could not be undone and required working with health system IT and financial departments and the pharmacy dispensing system software vendor to resolve the problem.

There were no additional changes to the documentation or communication process compared to 3-months post-CancelRx. An exemplar case from this time period is presented in Table 4.

**Table 4:** Exemplar medication discontinuation case 9-months post-CancelRx (pharmacist 7).

Task	Time period: 9-months post CancelRx
Receives	The pharmacist navigates to the “Inbound Communication Queue,” where new messages and prescriptions from prescribers are received. Several items are in the queue (indicated by rows in a table). They double click on a line/message, and the CancelRx screen appears.
Investigates	N/A
Matches	The CancelRx automatically matched the message to a prescription in the system. This is indicated because two rows of text are completed on the CancelRx screen. One row displays the received CancelRx message and the second row displays the associated prescription found in the pharmacy record.
Discontinues	The pharmacist clicks a button on the bottom left, “Remove from ICQ” [Inbound Communication Queue]. A pop-up appears asking, “Are you sure you want to remove from the inbound communication queue?” with the prompts “Yes” and “No.” The pharmacist clicks “Yes,” and the pop-up screen and CancelRx message disappear. The pharmacist is returned to the previous queue.
Documents	N/A
Communicates	N/A [not observed, but clinicians on the research team (PK, MS) confirmed that CancelRx automatically sends a notification back to the clinic regarding the successful discontinuation]

## 5 Discussion

Overall, introducing a novel health IT, CancelRx, changed the medication discontinuation workflow in community pharmacies. The functionality automated many tasks and increased the frequency with which medication discontinuation messages were communicated to the pharmacy (especially in the first three months after implementation). Within the literature, other methodologies have been used to describe and even quantify the impact of health IT on workflow (e.g., time-and-motion studies or time-on-task).^15^, ^16^, ^17^, ^18^, ^19^ In following those approaches, this study attempted to detail a linear and standardized medication discontinuation workflow. The observations from the pre-CancelRx period tended to fit this structure and aligned within the research team's mental model for medication discontinuation. After CancelRx implementation, however, the tasks still occurred but in a less structured order. Some tasks occurred simultaneously and instantaneously (i.e., CancelRx receiving, matching, discontinuing medications, and even communicating to the clinic), while others rarely happened, if at all (e.g., investigating and documenting). Even still, there was no one standardized, with some pharmacists insisting on spending more time researching CancelRx messages and others discounting much of the content without further investigation.

### 5.1 Medication discontinuation comparative workflow

One's first reaction to the change in workflow tasks over time may be to standardize further the medication discontinuation workflow, such as requiring profile reviews or investigation for all CancelRx messages. This applies a more comprehensive and positivist theoretical approach to identifying the one “true” way to receive and execute medication discontinuation messages, such as accomplishing all tasks in the predetermined order.^5^^,^^20^ Another approach, however, applying an interpretivist lens, suggests researchers consider how pharmacists are interacting with discontinuation messages and CancelRx. The interpretivist approach attempts to ascertain the meaning pharmacists gain from the messages and how that informs their actions. Two potential reasons for the observed changes in workflow between the 3- and 9-moth post CancelRx periods include: (1) pharmacists modified the CancelRx workflow through learned experience, and (2) pharmacists perceived the CancelRx messages as having “low-value.”

#### 5.1.1 Experience with CancelRx messages

In the case of CancelRx, pharmacists were the only individuals responsible for addressing and executing medication discontinuation messages, some receiving more than 10 over the course of a three-hour observation window. During the 3-months immediately following CancelRx implementation, the research team may have observed pharmacists acting as CancelRx beginners or novices. These pharmacists may have approached this new task with basic level understanding and used declarative knowledge or basic facts in their decision-making.^21^^,^^22^

The observed change in pharmacists' tasks over time may have been due, in part, to the repeated exposure to and experience with CancelRx messages. Decision-making literature suggests that as individuals gain expertise, they are better at detecting patterns and quickly retrieving relevant knowledge based on past experiences.^21^, ^22^, ^23^ This presents a possible explanation for why, at 9-months post-CancelRx, pharmacists clicked through the CancelRx messages and did not complete additional investigation tasks. This theory may suggest that as pharmacists became more comfortable with the CancelRx functionality, they recognized its ability to automatically discontinue medications within the computer and felt more comfortable quickly addressing the messages or placing them at a lower priority level amongst their other tasks.

Pharmacists incorporated and adapted to CancelRx within their already existing workflows. However, this adjustment is not always positive and becomes problematic when individuals become overburdened with a large proportion of messages they perceive to have little added value.

#### 5.1.2 Perception of “low-value” messages

Three months after CancelRx implementation, pharmacists were already citing frustration with the sheer number of messages received they perceived as “low-value.” In addition to solely gaining experience, pharmacists may have adapted their medication discontinuation process due to the perception that the CancelRx messages were simply another task providing little information and justifying quickly clicking through the prompts without truly reading or comprehending the message. This phenomenon has been termed “alert fatigue” and is often present for pharmacists when health IT applications present too many intrusive alerts that are mentally exhausting and time-consuming, causing them to ignore both relevant and irrelevant messages.^24^^,^^25^ Exposure to a large number of CancelRx messages considered low-value may cause pharmacists to click through all cases, even those that may truly warrant additional investigation or action.

In this case, the purpose of the CancelRx messages—communicating medication discontinuations to the pharmacy—is at least partially undermined because pharmacists are too overburdened to utilize its functionality. Two potential solutions exist to address this problem. First, organizations may choose to assess task responsibilities and consider the most appropriate pharmacy staff member to address CancelRx messages. Responsibility could be assigned to other staff members, such as technicians, who can systematically triage and address CancelRx messages based on their priority level. A technician may receive guidance and training when they can simply attest and remove “low-value” messages from the queue and leave the remainder for pharmacists to assess critically and problem solve. Previous studies have demonstrated the expanding roles of appropriately trained community pharmacy technicians, including tech-check-tech and technician vaccination initiatives.^26^, ^27^, ^28^ Pharmacy technicians demonstrated the ability to perform these delegated tasks accurately and safely, further supporting their involvement in CancelRx and medication discontinuation processes. Also, because CancelRx attempts to automatically match and discontinue prescriptions within the dispensing software, organizations may choose to eliminate the need for personal interaction or manual attestation and allow CancelRx to run completely behind-the-scenes (except for cases when the program is unable to match prescriptions).

Second, pharmacies may continue to require manual attestation, but the messages should be made more salient to pharmacists and their provision of patient care. This solution could involve health systems or prescribers no longer sending CancelRx messages in instances when they provide little added value to the pharmacy, such as one-time prescriptions for antibiotics or procedure preparation. Additionally, health systems and prescribers could include additional information to enhance message value, such as the reason for discontinuation (e.g., allergic response, adverse drug event, therapy change, or completion of therapy). The National Council on Prescription Drug Programs (NCPDP) supports this approach.^2^ Such information may be valuable to pharmacists to ensure appropriate changes are made to the patient's medication record or to monitor for future prescriptions (in the event of adverse drug events or allergic reactions). Additionally, providing this information within the CancelRx message may reduce the time needed for investigation tasks such as navigating to the dispensing record or EHR. These research findings illustrate the need for organizations to proactively assess and predict the impact of novel health IT on pharmacist work.^4^^,^^13^^,^^29^

## 6 Future research

Further research may include observations that follow the medication discontinuation message throughout the entire clinic and pharmacy workflows to verify the research team's process map (Fig. 2). This would include an assessment from the time a patient is seen in the clinic to how the medication is communicated to the pharmacy and canceled. Future studies may also assess the variation in CancelRx workflows across multiple pharmacy types and IT platforms. For example, this study utilized cases from pharmacies affiliated with an academic health system, but the workflow is likely different at national chain pharmacies or locally-owned independent platforms without a large network. Future evaluation of CancelRx should assess the occurrence and types of “low-value” or meaningless CancelRx messages and provide further guidance for CancelRx redesign and configuration. CancelRx redesign or health system configurations should maximize valuable communications to draw the user's attention to relevant messages, potentially providing systematic ways to triage high- and low-value messages that can be addressed by technicians or support personnel. Additional studies may assess the role of pharmacy technicians in the CancelRx and medication discontinuation process and the impact on pharmacist time and incidence of alert fatigue.

## 7 Limitation

As mentioned above, a limitation of the study is that the data collection took place within community pharmacies affiliated with a single health system (with access to an EHR). As a result, these findings may not be generalizable to pharmacies located outside of a health system (e.g., chain or independently owned community pharmacies). Additionally, the use of observations in the study made it difficult to collect descriptive statistics or additional information on the nature of the discontinuation messages received, such as the quantity and frequency of messages deemed “low value.” Finally, the pre-CancelRx and 9-month post-CancelRx observation periods occurred during the fall seasons, correlating to the Midwest influenza season. This meant that in addition to other dispensing responsibilities, the pharmacists and team members were also providing flu vaccines which may have influenced their ability to prioritize medication discontinuation messages.

## 8 Conclusion

Study results detail the change in the pharmacy medication discontinuation process before and after implementing CancelRx within one health system. Understanding the medication cancellation workflow reveals the benefits of CancelRx as an automated function and may further promote its widespread dissemination and acceptance. However, this study also exposed the importance of pharmacists' expertise, experience, and familiarity in the efficient and sustained use of CancelRx, and the possible experience of alert fatigue from an influx of messages with little perceived value. Such insights into the potential role of health IT can promote patient safety while at the same time prompting organizations to assess the perceived impact and benefit thoughtfully and proactively before the implementation of novel interventions.

## Declaration of Competing Interest

The authors declare that they have no known competing financial interests or personal relationships that could have appeared to influence the work reported in this paper.
